# Impact of Growth Rate on the Welfare of Broilers

**DOI:** 10.3390/ani14223330

**Published:** 2024-11-19

**Authors:** Anja B. Riber, Kaitlin E. Wurtz

**Affiliations:** 1 Department of Animal and Veterinary Sciences, Aarhus University, Blichers Allé 20, P.O. Box 50, DK-8830 Tjele, Denmark; 2 Livestock Behavior Research Unit, USDA-ARS, 270 S. Russel St., West Lafayette, IN 47907, USA

**Keywords:** activity level, breast muscle myopathies, broiler, broiler breeder, cardiovascular diseases, contact dermatitis, genotype, growth rate, walking ability, welfare

## Abstract

Selection for the more efficient production of broilers has resulted in rapid growth rates. The aim was to review the existing knowledge on the effect of growth rate on broiler welfare. Genotypes with faster growth rates consistently demonstrate poorer gait scores and increased prevalence of disorders affecting their legs than slower-growing genotypes. Reduced mobility places faster-growing broilers at an increased risk of developing contact dermatitis, as they spend increased durations sitting in contact with litter. Poor walking ability, heavy body weights, and conformational differences such as proportionally larger breast muscle in genotypes with faster growth can impact a bird’s ability to walk and navigate the environment, making it difficult to access resources and express natural behaviors. Faster growth has also been associated with poor cardiovascular health, increased susceptibility to heat stress, increased prevalence of mortality, ascites, as well as multiple breast muscle myopathies. Feed restriction, a practice associated with hunger and frustration, may be used to control the growth of broiler breeders, with birds having higher growth potential typically experiencing higher restriction levels. Overall, there is strong evidence that fast growth rates negatively impact welfare, and that slower-growing genotypes show significantly improved welfare. Furthermore, some evidence suggests that even minor reductions in growth rate can lead to welfare improvements.

## Simple Summary

Chickens raised commercially for meat production (known as broilers) have been intensively selected for greater muscle production and rapid growth, with growth increasing by over 400% from 1957 to 2005. However, animal welfare concerns regarding such rapid growth have been rising, and a transition to the raising of slower-growing chickens has been suggested as a potential solution. The aim of this review was to assess the existing scientific knowledge on the effect of growth rate on broiler chicken welfare. Overall, results from this review found that chickens with faster growth rates had increased prevalence of leg disorders, poorer ability to walk and perform various behaviors, increased prevalence of leg, skin, and cardiovascular disorders, increased susceptibility to heat stress, and higher mortality rates than chickens with slower growth rates. Therefore, it can be concluded that reductions in growth rate can lead to improvements in animal welfare.

## 1. Introduction

The industrialization of animal production has led to broiler chickens being heavily selected for production efficiency [1]. Modern commercial genotypes such as the Ross 308, Cobb 500, and Hubbard Flex have been bred to prioritize high feed efficiency, rapid growth rates, and large breast muscle sizes [2]. These genotypes can have growth rates of over 60 g/day and can reach slaughter weight in less than 6 weeks. However, this level of production efficiency does not come without consequences to welfare [3]. While it is true that environmental conditions and management practices, such as stocking density, nutrition, lighting conditions, and thermal conditions, can play a significant role in the welfare of broilers, studies continue to demonstrate that genetics, particularly those associated with growth rate, play the largest role [3,4].

Substantial evidence shows that birds selected for fast growth have reduced mobility [5,6,7,8]. This can restrict the performance of behaviors that they are motivated to perform and, in severe cases, limit their ability to access feed and water. In organic systems, where outdoor ranges and sometimes covered verandas are provided, birds may also be limited in their ability to use this space due to impaired mobility. Fast-growing birds are also less active and spend a considerable amount of time sitting in contact with litter [6,9,10,11]. Less litter turnover from reduced foraging behavior [12,13] can lead to a faster deterioration in litter quality, putting birds at an increased risk of contact dermatitis. During the aerobic breakdown of organic matter in the litter, ammonia and heat may be produced, contributing to poor air quality and increased thermal conditions [4,14]. Poor climatic conditions can put additional stress on the cardiovascular system which is already compromised in genotypes selected to prioritize the development of muscle over critical organs [15]. Death from cardiovascular conditions such as ascites or sudden death syndrome can significantly contribute to increased mortality in these genotypes [16]. On top of this, broiler breeders, the parent stock of broilers, are also at risk of compromised welfare due to the practice of feed restriction which can lead to chronic hunger and frustration [17]. Many of these conditions are interconnected and contribute to one another, but the common underlying contributing factor is the rapid growth that these birds experience.

**Figure 1:**
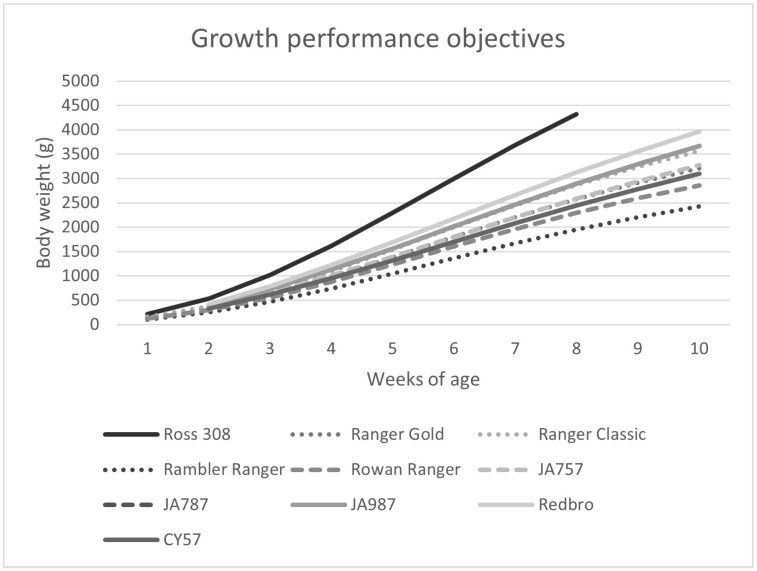
Plot of growth performance objectives of common commercial slower-growing genotypes compared to the fast-growing Ross 308 genotype from 1 to 10 weeks of age [20,21,22,23,24,25,26,27,28,29].

It has become increasingly difficult to ignore the welfare consequences associated with rapid growth and therefore there has been a growing push to select genotypes that have slower growth rates, such as those with growth rates of 35–50 g/day. Popular slower growing genotypes include the Hubbard JA757, JA787, JA987, CY57, and Redbro, the Aviagen Ranger Classic, Rambler Ranger, Rowan Ranger, and Ranger Gold, and the CobbSasso (Figure 1). Some producers are even raising dual-purpose genotypes in which the females go to egg production and the males are raised for meat [18]. These genotypes have much slower growth rates (e.g., <35 g/day).

Within the past decade, two reviews of the effects of growth rate on broiler welfare have been published. One focused on leg health and cardiovascular disease [3]. The other very recent review was structured according to housing conditions, age, and body weight of the genotypes compared and attempted to perform a quantitative analysis of effects of growth rate on mortality, gait score, footpad dermatitis, and hock burn, employing strict inclusion and exclusion criteria [19]. In contrast, the aim of this review was to conduct a comprehensive qualitative knowledge synthesis of the existing literature on the effect of growth rate on the welfare of broilers, including measures within all the major concerns of animal welfare.

## 2. Methods

### 2.1. Literature Search

A search for eligible studies was conducted using the databases Web of Science and Google Scholar using the search terms “broiler” and “growth rate” in combination with “gait score”, “walking ability”, “Bacterial chondronecrosis with osteomyelitis”, “tibial dyschondroplasia”, “varus valgus”, “contact dermatitis”, “footpad dermatitis”, “hock burn”, “activity”, “behavior”, “ascites”, “sudden death syndrome”, “breast muscle myopathy”, “wooden breast”, “white striping”, “deep pectoral myopathy”, “spaghetti meat”, “heat stress”, and “mortality”. Another search was performed using the terms “broiler breeder” with “growth rate” and “welfare”. The searches were conducted between June and August of 2023 and again in August 2024. Relevant citations found within the literature identified by the search were also included in this review.

### 2.2. Terminology

Throughout this review, the broilers with growth rates of over 60 g/day will be referred to as “fast-growing”, those with growth rates between 45 and 60 g/day as “intermediate-growing”, those with growth rates of between 35 and 45 g/day as “slower-growing”, and those with growth rates of less than 35 g/day as “slow-growing” [30]. When growth rates were not directly reported in the literature, final body weights were used to calculate growth rates assuming a day-old body weight of 45 g unless otherwise reported. In the few cases where neither growth rates nor body weights were available, the growth rate classification reported by the publication was used, with an indication that the true average daily gain is unknown.

## 3. Impact of Growth Rate on Welfare Indicators

The outcome of the literature search is structured and discussed in the following subsections according to the following welfare measures: (1) walking ability and leg health, (2) contact dermatitis, (3) activity and performance of preferred behaviors, (4) cardiovascular diseases, (5) breast muscle myopathies, (6) susceptibility to heat stress, (7) mortality rate, and (8) the impact of growth rate on the welfare of broiler breeders.

### 3.1. Walking Ability and Leg Health

It is well established that one of the primary risk factors associated with impaired walking ability is rapid growth. Poor walking ability is a condition with multiple etiologies which may be infectious or non-infectious (e.g., developmental, degenerative, or conformational) [31,32] and may be influenced by a number of factors including genetics, nutrition, and management practices [33]. Growth rate continues to be substantially linked with the development of leg disorders, with walking ability repeatedly being demonstrated to be poorer in broilers with faster growth [5,6,7].

Walking ability is commonly assessed through gait scoring, which is a measure (typically visual assessment) of an individual’s ability to walk. Several gait scoring protocols exist, but the system commonly referred to as the Bristol scale is a validated method (see [34]), which is often used as the gold standard for assessing walking ability and for the validation of novel or alternative methods used for the assessment of walking ability. The Bristol scale ranges from a score of 0 (GS0) where the bird walks with no detectable abnormality, up to a score of 5 (GS5) where the bird is unable to walk [35]. Poor gait scores can result from a multitude of conditions but do not provide a clear indication of the specific etiology. Poor gait scores are considered a major welfare problem within the broiler industry and can impact behavior, ability to access resources such as food and water, are linked with increased morbidity and mortality rates, and may be associated with pain [32,36,37,38,39]. Poor walking ability is heavily associated with fast growth [33,40,41] and associated changes in conformation such as larger breast muscles, which can lead to birds modifying their gait to achieve balance [42].

There are extensive data linking rapid growth rates with poorer gait scores gained through surveys of commercial flocks and experimental trials (Table 1). Tahamtani et al. [5] conducted a large survey of conventional (Ross 308; ADG: 63 g/day) and organic (JA757 and CYJA57; max. ADG: 35 g/day) broilers in Denmark between November 2016 and April 2017 and found that the slow-growing genotypes housed under organic conditions had 9.8% prevalence of gait scores ranging from 2 to 5 (moderate to severe gait defects) compared to 36.2% in the conventional systems with a fast-growing genotype when assessed prior to slaughter. However, these differences could in part be attributed to housing conditions. A more recent survey conducted between June and November 2022 in Denmark found a substantial deterioration in walking ability from the previous survey in both organic and conventional broilers, with an eightfold decrease in frequency of GS0 and a two-fold increase in gait scores ranging from 1 to 3 in organic production [43]. The authors speculated that this change in walking ability may be attributed to changes in genotype used and allowed daily growth rates. Since the last survey, the genotype used in organic production has changed from JA757 and CYJA57 to Ranger Gold. Furthermore, the allowed daily growth rate increased from 35 to 38 g/day. Dixon [6] compared the gait scores of an intermediate-growing genotype (Hubbard JA757; ADG: 46 g/day) with three fast-growing genotypes (Cobb500, JA308, and Hubbard Flex; ADG: 63 g/day) all housed under similar experimental conditions. The prevalence of fast-growing birds with obvious lameness ranged from 27 to 39% compared to 10% in the intermediate-growing genotype. In another study, where a fast-growing genotype (ADG: 63 g/day) was compared to two intermediate-growing genotypes (ADG: 45 and 49 g/day) housed under the same experimental conditions, it was found that the fast-growing genotype had substantially worse gait scores, greater culls due to leg issues, and greater postmortem rejections [7]. When comparing between the two intermediate-growing genotypes in this study, the genotype with a growth rate of 45 g/day had a greater proportion of birds with gait scores 0 and 1, and a smaller proportion of birds with gait scores of above 2 than the genotype with a growth rate of 49 g/day [7]. Recently, a study comparing the walking abilities of two intermediate-growing genotypes (ADG: 51 and 47 g/day) housed under similar commercial conditions found the slowest growing of the two genotypes to have a lower occurrence of gait scores known to be associated with reduced welfare, i.e., gait scores of 2–5 on the Bristol scale [8]. In an experimental study comparing six genotypes differing in growth rates, the fast-growing Ross 308 genotype (ADG: 61 g/day) had a substantially lower prevalence of normal gait than the four intermediate-growing genotypes (Redbro, ADG: 49 g/day; Rustic Gold, ADG: 48 g/day; Ranger Classic, ADG: 47 g/day; Hubbard JA787, ADG: 46 g/d) and a slower-growing genotype (Hubbard JA757, ADG: 42 g/day) [44]. The Rustic Gold had a lower prevalence of normal gait than the three slowest growing of the other genotypes, i.e., the Ranger Classic, Hubbard JA787, and Hubbard JA757.

Kestin et al. [40] explored the relationship between growth rate and gait scores across 13 different genotypes with a wide range of growth potential, including fast-growing, intermediate-growing, slower-growing, and traditional ‘dual-purpose’ genotypes, fed two different diets (non-limiting or a Label Rouge diet which was lower in protein and energy). Irrespective of genotype, this study found a clear relationship between liveweight and gait scores, with birds becoming linearly more lame the heavier they became, over a threshold of 1.25 kg. When correcting for liveweight differences, the authors found a significant relationship between the rate of growth and lameness. For example, birds reaching 2.7 kg in 54 days (ADG: 49 g/day) had one gait score unit worse (when scored on a 3 point scale [35]) than birds reaching the same weight in 81 days (ADG: 33 g/day). A similar effect was found in a study of the Cobb 700 genotype where reducing growth rate by the use of feed restriction resulted in improved gait score (ADGs: 48 g/day vs. 66 g/day) and bone strength (ADGs: 48 g/day vs. 60 g/day) [46]. In a study comparing Ross 308 broilers with an average daily weight gain of over 60 g/day with Rowan Rangers, an intermediate-growing genotype (ADG: around 50 g/day), the Ross 308 birds had significantly poorer walking ability, which rapidly deteriorated from 6 weeks of age and onwards [48]. At 6 weeks of age, the proportion of birds with gait scores ranging from 2 to 5 (scores in this range are known to cause changes in behavior [39]) was 8.8% and 0.3% for the Ross 308 genotype versus the Rowan Ranger genotype, respectively. The Ross 308 birds also had a greater percentage of culls due to leg issues [48].

Latency to lie (LTL) tests (a measurement of how long a bird can remain standing) and obstacle tests (a measure of a bird’s ability to navigate physical barriers to access a reward such as food) are two alternative ways of assessing walking ability other than traditional gait scoring. Santos et al. [49] employed both tests on 16 different genotypes with various growth rates (ADG: 20–69 g/day) and found that the slowest growing birds had the longest LTL times and the genotypes with growth rates between 44 and 48 g/day had a higher number of obstacle crossings than the genotypes with growth rates between 50 and 69 g/day, suggesting that slower growth was associated with better leg health. When comparing genotypes within similar growth rate groups (“Slow”: 44–48, “Moderate”: 50–51, “Fast”: 54–55, “Conventional”: 66–69 g/day) there were no significant differences between genotypes for neither the LTL nor the number of obstacle crossings.

Bacterial chondronecrosis with osteomyelitis, tibial dyschondroplasia, and varus valgus disease are believed to be the most common disorders affecting leg health in broilers [32,50,51]. Bacterial chondronecrosis with osteomyelitis (BCO), previously referred to as femoral head necrosis, represents a major cause of impaired walking ability [51]. This condition is a bacterial infection which infects the skeletal system and often progresses to necrosis of rapidly growing bones subjected to mechanical stress [52]. Birds with faster growth rates are at an increased risk of infection due to a combination of impaired immune response [53,54] and microfractures in the cartilage and bone due to rapid growth and increased stress on the developing skeletal structure, creating a suitable environment for bacterial colonization [51,52]. Multiple studies have demonstrated evidence for a genetic component to the susceptibility of developing BCO [55,56]. Wideman, et al. [57] studied four commercial crosses of broilers of which two were standard crosses with fast growth at an early age (ADG at 56 days: 72 and 74 (males), 61 and 63 (females)) and two were high-yield genotypes with slower initial growth (ADG at 56 days; 71 and 71.5 (males) and 60 (females)). The birds were reared on wire floors, a condition known to trigger BCO due to imposing a rigorous, sustained mechanical and physiological challenge. The genotypes with fast growth at an early age were found to be more susceptible to developing lesions characteristic of BCO and had higher incidences of lameness. Additionally, across all commercial crosses examined in this study, the female birds had lower incidences of lameness, likely attributable to their slower growth than their male counterparts [57]. In another study, incidences of BCO in broilers were reduced by restricting their feed intake to 60% of ad libitum intake [58]. This evidence supports the hypothesis that rapid early growth predisposed birds to the development of BCO.

Intense selection pressure over the years has contributed to an increased prevalence of developmental disorders, especially those linked to growth rate and the development of the musculoskeletal system. Tibial dyschondroplasia (TD) is a condition which is associated with rapid growth and is characterized by an abnormal mass of cartilage in the proximal end of the tibia, resulting from inadequate vascularization and mineralization of the growth plate and bones [59]. This abnormal mass can cause angular and rotational abnormalities in the leg and hip, which can contribute to impaired walking ability, though the association between TD and gait score may depend on the severity of TD. Prevalence estimates vary widely between studies, and estimates have ranged from high proportions such as 57% as reported in a Danish study [60] to only 9.2% in an experimental flock located in the United States [61], though genetic selection against TD has resulted in a lower prevalence in modern-day flocks [5,31,62]. In a study by Kestin et al. [63], four different commercial crosses with small but significant differences (less than a gram) in growth rates (relative growth rate from 21 to 30 days: 58 g/day and from 28 to 35 days: 28 g/day) were examined for susceptibility to various leg health traits, including TD. Significant differences were found between the genotypes in walking ability, TD, and angulation of the hock joint, suggesting that there is a genetic component to TD, though association with growth rate was not clear. Shim et al. [50] examined prevalence of TD in two subpopulations of mixed-sex Arkansas random-bred broilers and found that those with faster growth (top quarter of the population; ADG: 45 g/day) from hatch to six weeks of age had a significantly higher incidence of TD than those with growth rates in the bottom quarter (ADG: 33 g/day) of the population. Similarly, Fanatico et al. [64] found that bird genotype had a greater effect on the prevalence of TD incidence than production system or diet, with lower prevalence of TD being observed in flocks of a slow-growing genotype (ADG: 25 (outdoor access) and 23 (indoor only) g/day) than an intermediate-growing genotype (ADG: 53 (outdoor access) and 54 (indoor only) g/day).

Varus valgus deformity (VVD) is a developmental disorder resulting from rapid weight gain and inadequate nutrition which causes either an inward (varus) or outward (valgus) twisting of the legs [60]. Angulations of the leg can vary in severity, ranging from mild (10–25 degrees) to over 45 degrees in severe cases [50]. Due to the abnormal pressure on the deformed legs, birds with VVD are at an increased risk of developing additional conditions including slipped tendons or leg fractures. In the study mentioned above, Shim et al. [50] found a significantly higher incidence of valgus for both legs in the subpopulation with more rapid growth (ADG: 45 compared to 33 g/day). Another study found a reduction in the occurrence of varus-valgus deformities when the growth of broilers was slowed down (ADG: 47 g/day) by feeding a low energy diet compared to those fed a high energy diet (ADG: 52 g/day) [65]. The authors hypothesized that the improvement in tibial angle may have resulted from better quality tendons and ligaments as a result of more frequent locomotor activity in birds fed a reduced energy diet in the form of finely ground particles [65,66]. An additional study by Classen and Riddell [67] similarly found that angular bone deformities can be reduced by slowing growth (by altering photoperiod) in the first 15 to 20 days of life.

It is clear from the available evidence that there is an effect of genotype on leg health and walking ability. Genotypes with more rapid growth rates are more likely to suffer from poor leg health. Rapid growth, especially at a young age, places additional stress on an underdeveloped skeletal system. This can increase the risk of developing various leg health disorders. Additionally, many genotypes that have been selected for rapid growth have also been selected for changes in muscle composition, which can negatively impact walking ability as birds need to alter their gait to maintain balance. When altering growth rate through changes in diet, slower growth rates even within a genotype have been shown to improve walking ability. Body weight in general has also been shown to impact leg health, with lighter birds having improved walking ability than heavier birds, however, when correcting for live weight it appears that rate of growth still has a substantial impact. Even minor differences in growth rates have been shown to lead to significant differences in walking ability. While genetic selection has led to improvements in some leg health traits, such as a reduction in the prevalence of TD, recent surveys of broiler flocks are still indicating a link between leg health and genotype, with faster growth leading to poorer outcomes. Walking ability is a critical measure to consider due to its association with various welfare indicators.

### 3.2. Contact Dermatitis

Broilers with poor mobility are less active and are more likely to spend time in a sitting position with their hocks and breast in contact with the litter. Their reduced engagement in foraging activity could also contribute to a faster deterioration in litter quality as there may be a lower turnover of the litter [12,13]. Prolonged contact with deteriorating litter puts birds at a higher risk of developing contact dermatitis on their feet (footpad dermatitis), hocks (hock burn), and breast (breast burn) [68]. Early in development, lesions may be small and superficial, appearing as mild discoloration or a rough abrasion, but can develop into larger wounds, and if the skin barrier is broken down, they may provide a gateway for infection [69]. Leg issues have been shown in some studies to be significantly correlated with increased footpad dermatitis and increased hock burn [70,71].

Given the link between rapid growth rates and poor gait scores, it is not surprising that studies have shown a link between rapid growth and a higher prevalence of contact dermatitis. Dixon [6] compared the extent of footpad dermatitis and hock burn severity between an intermediate-growing genotype (Hubbard JA757; ADG: 46 g/day) with three fast-growing genotypes (Cobb500, JA308, and Hubbard Flex; 63 g/day) all housed under similar experimental conditions and found that the Hubbard JA575 genotype had a lower prevalence of hock burns than the other genotypes; however, no differences in footpad dermatitis scores were observed. Santos et al. [49] assessed the incidence of contact dermatitis in 14 genotypes differing in a growth rate at two target weights (2.1 and 3.2 kg) and found that at 2.1 kg, the genotypes with growth rates of 66 and 69 g/day had greater incidences of footpad dermatitis than genotypes with growth rates of 54 to 55 and 50 to 51 g/day. At 3.2 kg, the genotypes with growth rates of 66 and 69 g/day had greater incidences than all other genotypes. At 2.1 kg, genotypes with growth rates from 66 to 69 and from 50 to 51 g/day had greater occurrences of hock burn than those with growth rates from 44 to 48 g/day. At 3.2 kg, genotypes with growth rates from 66 to 69 and from 54 to 55 g/day had more incidences of hock burn than genotypes with growth rates from 50 to 51 and 44 to 48 g/day. Within the growth rate categories there were no differences among genotypes at both target weights assessed. In a study using register data from nine years of broiler production in the Netherlands, Slegers et al. [72] found a lower occurrence of footpad lesions in slow- and intermediate-growing genotypes than in fast-growing genotypes, and in slow-growing genotypes compared to intermediate-growing genotypes (ADGs: not reported). In contrast, Guinebretière et al. [44] found more inconsistency in the link between growth rate and footpad dermatitis in an experimental study comparing six genotypes. The fast-growing Ross 308 genotype (ADG: 61 g/day) had a lower prevalence of feet not affected by dermatitis than two intermediate-growing genotypes (Rustic Gold, ADG: 48 g/day; Ranger Classic, ADG: 47 g/day) and a slower-growing genotype (Hubbard JA757, ADG: 42 g/day), but did not differ from two other intermediate growing genotypes (Redbro, ADG: 49 g/day; Hubbard JA787, ADG: 46 g/d). The prevalence of hock burn was not affected by growth rate.

Kjaer et al. [73] followed the development of footpad dermatitis and hock burn in Ross 308 (fast-growing: ADG: not reported) and a slow-growing dual purpose genotype (ADG: not reported) from 8 days of age until slaughter. Footpad dermatitis was not detected in the dual-purpose genotype but began at 2 weeks of age and increased until slaughter at 6 weeks in the Ross 308 genotype. Hock burn was observed in both genotypes but remained very rare until around 4 weeks of age where the incidence increased sharply for the fast-growing genotype. Rayner et al. [7] similarly found higher levels of hock burn in a fast-growing genotype (ADG: 63 g/day) compared to two intermediate-growing genotypes (ADG: 45 and 49 g/day). These fast-growing birds also had higher levels of footpad dermatitis. There were no significant differences found in hock burn nor footpad dermatitis scores between the two intermediate-growing genotypes (ADG: 45 and 49 g/day) in this study [7]. In a study conducted by Wilhelmsson et al. [48], a higher proportion of Ross 308 birds (fast-growing; ADG: over 60 g/day) had footpad dermatitis and hock burn compared with Rowan Ranger birds (intermediate-growing; ADG: about 50 g/day) throughout the rearing period, and increased to a greater extent in the Ross 308 birds over time. Steenfeldt et al. [74] found significant differences in footpad dermatitis at the time of slaughter between the two Ross genotypes, the fast-growing 308 (ADG: 61 g/day) and a slow-growing Ross genotype derived from 1972 (ADG: 24 g/day), with only three out of seven hundred and twenty-two of the slow-growing genotype showing signs compared to 67.2% of the fast-growing genotype. Allain et al. [75] measured lesions at the slaughter plant and found that an intermediate-growing genotype (ADG: 45 g/day) had deeper footpad lesions than a slow-growing genotype (ADG: 34 g/day). Yamak et al. [76] compared a fast-growing genotype (Ross 308; ADG: 70 g/day) with two intermediate-growing genotypes (ROSS × RIR and ROSS × BAR; ADG: 49 and 48 g/day, respectively) and found that the fast-growing birds had significantly higher footpad dermatitis scores at both 6 and 7 weeks than both of the intermediate-growing genotypes, and the genotype with a growth rate of 49 g/day had a higher prevalence of footpad dermatitis than the genotype with a growth rate of 48 g/day. Under organic conditions, Ross 308 (ADG: 55 g/day) birds were found to have higher frequencies of footpad dermatitis than slow-growing genotypes (ADG: 16–31 g/day), with 60% of the Ross 308 birds having a severe footpad dermatitis score compared to 20% of the Naked Neck (ADG: 31 g/day) genotype and 30% of the Kabir (30 g/day) genotype [77]. The remaining genotypes investigated (ADG: 16, 17, 23, 25, and 27 g/day) had no instances of footpad lesions.

Based on the available evidence, it is apparent that the prevalence of contact dermatitis is greater in flocks with faster growth rates. This is likely a result of a combination of leg issues and poorer litter quality in fast-growing genotypes. In studies that assessed contact dermatitis throughout life, it appeared that the prevalence of footpad dermatitis and hock burn were low early in life (i.e., before 4 weeks of age) but increased more rapidly and to a greater extent in the faster growing genotypes later in life. The results from studies of genotypes with minor differences in growth rates (4 ± 3 g) are inconsistent. Some studies have found that minor differences in growth rates result in a similar prevalence of contact dermatitis ([77]; 16, 17, 23, 25, 27 g/day); [7]), whereas other studies have found that a minor reduction in growth rate leads to a lower prevalence of hock burns [49] and footpad dermatitis ([77]; 27 vs. 30–31 g/day); [76]).

### 3.3. Activity and Performance of Preferred Behaviors

Modern day chickens retain a similar behavioral repertoire as their ancestors, the red junglefowl [78], and the evidence suggests that internal motivational drivers to perform these behaviors are preserved [79]. Rapid growth and heavy body weights can negatively impact a bird’s ability to walk and navigate their environment, making it difficult to express behaviors such as standing, foraging, exploring, perching, and roosting on elevated structures [6,11,80,81]. Pain associated with rapid growth may also impact a bird’s behavioral repertoire resulting in lower activity and less performance of behaviors associated with positive affective states such as comfort behaviors [39,82]. Broilers exhibit comfort behaviors such as preening, dustbathing, wing flapping, and leg stretching [83]. The inability to perform comfort behaviors is considered a major welfare concern [84]. Poor walking ability can negatively influence a bird’s ability to perch [39]. Impaired cardiovascular function may also impair birds’ ability to perform motivated behaviors, as chronic heart failure will result in hypoxemia and conditions, such as ascites, causing fatigue and discomfort [85]. Inability to perform motivated behaviors may result in frustration [32,86] or fear, such as when unable to perform anti-predatory behaviors such as roosting [87,88]. Contrary to their wild counterparts that spend a substantial proportion of their time budget foraging and exploring and only roughly 10% of their day sitting [89], fast-growing broilers are generally inactive, sitting as much as 70–80% of the day [6,9,90].

Dawson et al. [10] examined activity, behavior, and enrichment use of 14 broiler genotypes with different growth rates: two conventional fast-growing genotypes (ADG: 66–69 g/day) and twelve intermediate-growing genotypes with average daily gains ranging from 44 to 56 g/day. All genotypes were housed under the same experimental conditions. Overall, the faster-growing genotypes were more inactive, spent more time sitting and feeding, spent less time standing and walking, and used the enrichments less than the slower-growing genotypes. These differences between genotypes with different growth rates were most apparent at younger ages. At older ages (around 8 weeks), all genotypes displayed similar high levels of inactivity (78–80% of the day). However, slower-growing genotypes were older than the fast-growing genotypes when reaching the highest levels of inactivity (8 weeks vs. 6 weeks). In general, genotypes with similar growth rates demonstrated similar activity and time budgets with one another, however there were a few exceptions. Amongst the genotypes with the slowest growth rates, differences in time spent walking were observed at 26 days of age with one genotype (ADG: 44 g/day) performing significantly less walking behavior than a genotype with a growth rate of 46 g/day. However, the other two genotypes within this growth rate group (ADG: 44 and 48 g/day) did not perform significantly different amounts of walking. The differences observed at 26 days of age were not observed at days 42 and 56 of the experiment. Within categories of genotypes with similar growth rates there were differences in enrichment use, however the authors did not find any consistent pattern with respect to genotype or specific enrichment type. The authors concluded that there may be aspects in addition to growth rate that impact behavior such as temperament, genotype-specific growth curves, or differences in body conformation. Somewhat similar results were found in an experimental study comparing six genotypes differing in growth rates [44]. The fast-growing Ross 308 genotype (ADG: 61 g/day) was overall more inactive, perching less, and interacting less with environmental enrichment than the four intermediate-growing genotypes (Ranger Classic, ADG: 47 g/day; Redbro, ADG: 49 g/day; Rustic Gold, ADG: 48 g/day; Hubbard JA787, ADG: 46 g/d) and a slower-growing genotype (Hubbard JA757, ADG: 42 g/day). The slow-growing genotype performed more pecking and foraging than the other genotypes. Zhou et al. [46] found that reducing the growth rate of the fast-growing Cobb 700 genotype (ADG: 66 g/day) by the use of feed restriction to intermediate growth (ADG: 48 g/day) resulted in an increased activity index. However, this may be caused by the hunger experienced by the feed restricted birds, resulting in restlessness and increased time spent searching for food.

When comparing time spent foraging and walking, Bokkers and Koene [81] found that a slow-growing genotype (Hubbard JA 656; ADG: 25 g/day) perched, walked, and scratched more than an intermediate-growing genotype (Hubbard HI-Y; ADG: 51 g/day). The intermediate-growing genotype spent more time sitting, feeding, and drinking than the slow-growing genotype. This study concluded that both intermediate- and slow-growing genotypes are motivated to perform certain behaviors, however the ability to perform them becomes hampered with increasing age (and associated increase in weight). When housed to 12 weeks, the intermediate-growing genotype performed increased preening behavior. Preening behavior is a comfort behavior, but if excessively expressed, it may have developed into a displacement behavior indicating frustration [91].

Wallenbeck et al. [11] similarly found decreased activity and foraging behavior as an intermediate-growing (Ross 308; ADG: 56 g/day) genotype and a slower-growing (Rowan Ranger; ADG: 40 g/day) genotype aged. In this study, the Ross 308 birds were less active, sat more, fed and drank more frequently, and perched less during the daytime than the slower-growing genotype. Abeyesinghe et al. [92] found more side-lying, less walking, and less perching in a conventional fast-growing genotype (ADG: 66 g/day) versus two intermediate-growing genotypes (ADG: 49 and 46 g/day). Dixon [6] found that an intermediate-growing genotype (Hubbard JA757; ADG: 46 g/day) spent more time being active, and less time sitting, feeding, and drinking than three fast-growing commercial genotypes (Ross 308, Cobb 500, and Hubbard Flex; average ADG: 63 g/day). In a study examining the resting and perching behavior of two intermediate-growing genotypes (Aviagen Rowan Ranger and Hubbard CYJA57; ADG: 45 g/day) versus the fast-growing Ross 308 genotype (ADG: 72 g/day), it was found that resting frequency did not differ between the three genotypes, however the fast-growing birds perched less during the day and night and only utilized the lowest perch level, whereas the intermediate growing genotypes utilized all three heights [93]. Malchow et al. [94] compared an intermediate-growing (Ross 308; ADG: 59 g/day) with two slow-growing (Lohmann Dual: ADG: 31 g/day; Lohmann Brown Plus: ADG: 20 g/day) genotypes and found that Ross 308 birds reduced their use of elevated structures during the day period from the 3rd to 4th week of age. Over time, the activity level decreased for all genotypes, but the decrease was most substantial for the Ross 308 compared to the two slow-growing genotypes.

Use of provided enrichment materials may be hindered by mobility issues related to rapid growth. When straw bales were provided, Rayner et al. [7] observed more slower-growing birds (two genotypes with intermediate growth: 49 and 45 g/day, respectively) on top of the bales than the fast-growing genotype (ADG: 63 g/day). The fast-growing genotype also displayed lower levels of behaviors indicative of positive welfare including play and ground scratching. During a qualitative behavior assessment (QBA), the genotypes with intermediate growth displayed more “happy/active” scores (not defined, which is a general condition in the QBA method). No significant differences in behavior were observed between the genotypes with growth rates of 45 and 49 g/day suggesting that the slightly faster growth rate did not impair the birds’ ability to perform these specific positive behaviors.

Castellini et al. [77] assessed the adaptability of genotypes with various growth rates to an organic rearing system. Genotypes were categorized by their growth rates by the authors as follows: Group 1 with the slowest growth (Ancona, ADG: 17 g/day; Leghorn, ADG: 16 g/day; Cornish × Leghorn, ADG: 23 g/day), Group 2 (Kabir, ADG: 30 g/day; Naked Neck, ADG: 31 g/day; Robusta Maculata, ADG: 27 g/day; Gaina, 25 g/day) and Group 3 with the fastest growth (Ross 308; ADG: 55 g/day). At 11 weeks of age, initial interest (first 5 min of the presence) shown by the birds towards the observer, time spent on the range, and distance ranged from the shelter were assessed. The genotypes with the slowest growth rates showed higher initial interest in the observer, spent more time outdoors, and ranged further from the shelter, exploiting the range to a greater extent. The Ross 308 birds showed the lowest initial interest, spent the least amount of time outdoors, and did not range as far on the range. The genotypes in Group 2 performed intermediately compared to the slowest- and fastest-growing genotypes. An effect of genotype was found on fear levels assessed with a tonic immobility (TI) test, with the Ancona, Leghorn, and Robusta Maculata showing the shortest TI durations suggesting they were less fearful than the Kabir, Gaina, and Ross genotypes which had the longest TI durations. However, as the test was performed at the same age (11 weeks) as opposed to the same body weight, this result may have been confounded with effects of body weight, as a heavy body weight may reduce the capacity of a broiler to right itself, increasing the TI duration.

While fear levels are likely a contributing factor to extent of range use, there is the possibility that mobility and general activity levels, which are closely linked with growth rates, could influence birds’ ability to access the outdoor space. Nielsen et al. [95] found that a slow-growing genotype (Labresse Cross; ADG: 31–33 g/day) used the outdoor area more than an intermediate-growing genotype (Ross 208; ADG: 45–46 g/day). The Ross 208 birds in this study had worse gait scores. In addition, 3.9% of the birds were found at slaughter to have deep pectoral myopathy (see more in Section 3.5. “Breast muscle myopathies”), which may have contributed to the lower range use.

Overall, given the evidence it appears that slower-growing genotypes have more freedom of movement and thus a greater ability to perform motivated behaviors. In the literature, faster growth rates are consistently associated with birds spending more time sitting and less time walking, foraging, perching, engaging with enrichment, and engaging in behaviors associated with positive affective states such as dustbathing and play. The differences in behavioral time budgets were most apparent at younger ages. At older ages, the slower growing genotypes may eventually display similar levels of inactivity as the faster growing genotypes but take longer to reach the same levels. In general, genotypes with similar growth rates displayed similar behavioral patterns and where differences did exist, there was not a clear relationship with growth rate. When it comes to the use of an outdoor range, there is evidence that the slower the growth rate, the more likely they are to utilize the range area and to range further from the house. Within the same growth potential there may be differences in range use between genotypes, but research is too sparce to draw conclusions, especially since there are many factors known to impact range use.

### 3.4. Cardiovascular Diseases

Selection for rapid growth in broilers has favored the growth of muscle, causing bone and organ development to lag behind. Proportionately, the heart and lungs of a fast-growing genotype (Ross 308) are smaller than in slower-growing genotypes (Athens-Canadian Random Bred Control, a genotype developed in the year 1957) [96]. Larger muscles have an increased oxygen demand, causing further stress on an already compromised cardio-respiratory system which can lead to cardiovascular conditions such as sudden death syndrome (SDS) and ascites. Birds which succumb to SDS often show heart abnormalities such as heart arrhythmias. In flocks of fast-growing broilers, 17–35% of the broilers have been estimated to be affected by cardiac rhythm disturbances, though these estimates are from 25 years ago [97,98]. The prevalence increased with increasing age and was higher in males than females. When fast-growing genotypes were subjected to feed restriction of approximately 60% ad libitum to slow down their growth, the prevalence of arrhythmia was reduced to less than 2% [85]. In slow-growing genotypes, such as leghorns, it is believed that the prevalence is less than 1% [85].

Mortality from ascites has been estimated to account for 5–8% of total mortality, with estimates as high as 30% in flocks of heavier (e.g., >3 kg) broilers [16,99,100]. When Rayner et al. [7] examined the percentage of birds rejected at slaughter due to ascites, they found that 0.16% of fast-growing birds (ADG: 63 g/day) were rejected, whereas no birds of the two intermediate-growing genotypes were (ADG: 45 and 49 g/day). Selection against ascites seems to have been successful as the higher prevalence was found in the older literature. As with SDS, death from ascites may similarly be reduced by slowing weight gain through restriction of feed [101]. Based on the available evidence, it is suggested that both SDS and ascites may virtually be eliminated through the use of slower-growing genetics, especially when in conjunction with improved environmental conditions such as lower stocking densities and improved lighting schemes [102].

### 3.5. Breast Muscle Myopathies

Multiple breast muscle myopathies have emerged with the development of fast-growing genotypes of broilers. These include deep pectoral myopathy (DPM; also known as “green muscle disease”, pale-soft-and-exudative (PSE)-like meat, wooden breast syndrome, white striping, and “spaghetti meat” [103]. Birds afflicted by these conditions may experience muscle weakness and long-lasting discomfort, though evidence regarding the welfare impacts of these conditions is lacking. One study found a correlation between impaired walking ability and the presence of wooden breast syndrome [104], whereas another study found no association between broilers with slight gait impairments (gait scores 1–2) and wooden breast syndrome [105]. It is believed that heavy selection for fast growth has made broilers more susceptible to these conditions, due to the placement of unsustainable pressure on muscle metabolism, triggering degenerative features which may lead to muscular conditions [106,107,108]. In addition to high growth rates, there is evidence that heat stress, which fast-growing broiler genotypes are more susceptible to, may induce muscle damage like PSE at higher rates [109,110].

Wooden breast is characterized by hard, bulging muscle tissue resulting from necrotic muscle fibers that have been replaced with connective tissue. This condition is often accompanied by white striping, which is characterized by visible white striations which run parallel to the muscle fibers [111]. Multiple studies have reported a higher prevalence of white striping in flocks with higher growth rates [108]. In a study aiming to directly assess the impact of growth rate on the prevalence of white striping, researchers fed an intermediate-growing broiler genotype (Cobb 500) either a low- (ADG: 50 g/day) or high-energy (ADG: 55 g/day) diet [112]. The results from their study suggested that an increased growth rate of 5 g/day resulted in an increased occurrence of severe white striping in broiler breast fillets. In a study comparing an intermediate-growing genotype (Hubbard JA757; ADG: 46 g/day) with three fast-growing genotypes (Ross 308, Cobb 500, Hubbard Flex; ADG: 63 g/day), the intermediate-growing birds were found to have lower levels of white striping and wooden breast than the other genotypes [6].

Breast muscle myopathies also appear to be more prevalent in high-yielding genotypes, which are likely to also have faster growth rates due to overall selection for more efficient production. For example, white striping [112,113,114,115], deep pectoral myopathy [114], wooden breast [114], and spaghetti meat [115] have been observed in higher frequencies in genotypes selected for high yield, though their relationship with growth rates remain unclear as there are likely other factors, such as body conformation or muscle size, which may contribute to the development of these conditions.

### 3.6. Susceptibility to Heat Stress

Fast-growing genotypes produce more heat compared to slower-growing genotypes due to their increased metabolism, making them more susceptible to stress from high temperatures [116]. Data on the susceptibility of various broiler genotypes are currently limited; however, there is some evidence suggesting that the higher the growth rate the more susceptible the birds will be. For instance, Deeb and Cahaner [117] found a positive association between growth rate and the reduction in body weight and body weight gain at high environmental temperatures (avg. 30 °C), meaning that the higher the growth rate potential, the more it was negatively influenced by high environmental temperatures. Likewise, Cahaner and Leenstra [118] compared five genotypes with different weight gain potentials (ADG: 28, 29, 30, 31, and 33 g/day) and found that the growth reduction due to high environmental temperature (32–33 °C) was largest in groups with the highest growth rate. Berrong and Washburn [119] compared a slower-growing population (Athens-Canadian Random Bred; ADG: not reported) of broilers to faster-growing broilers (ADG: not reported) and found that decreased body weight gain was more severe and mortality was higher in the faster-growing broilers in response to heat stress. Steenfeldt et al. [74] also compared a fast-growing (Ross 308; ADG: 61 g/day) genotype to a slow-growing genotype (Ross derived from the year 1972; ADG: 24 g/day) and found that while activity was higher and panting levels were lower in the slow-growing genotype, these results were not impacted by the temperature treatment (warm: 21 °C or cold: 15 °C). Yalçin et al. [120] found that a slower-growing genotype (ADG: 43 g/day) had relatively lower mortality rates and body temperatures than two intermediate-growing (ADG: 47 and 48 g/day) genotypes. Compared to the Red Jungle Fowl (ADG: 6 g/day), a faster-growing indigenous village fowl from Malaysia (ADG: 10 g/day) and a slower-growing commercial broiler chicken (Ross; ADG: 42 g/day) were found to be more susceptible to acute heat stress [121].

### 3.7. Mortality Rate

Following the lower prevalence of life-threatening conditions, broilers with a lower growth rate potential generally demonstrate lower mortality rates than fast-growing genotypes, having both a lower number of dead birds found and number of culls (Table 2). Reduced mortality rates in slower-growing broilers can likely be attributed to their improved cardiovascular health, lower instances of contact dermatitis (i.e., gateways to potentially fatal secondary infections), lower susceptibility to heat stress, and improved walking ability allowing them to reach life-sustaining resources. The improved ability to perform highly motivated behaviors may also indirectly lead to improved health outcomes which could reduce mortality risk. For example, as mentioned in Section 3.2, an increased foraging activity slows down the deterioration in litter quality due to the higher turnover of litter [12,13]. This in turn reduces the risk of footpad dermatitis and associated secondary infections which may be fatal [71]. Improved overall health further reduces the number of birds that would need to be culled on the farm. Dixon [6] found that an intermediate-growing genotype (Hubbard JA757; ADG: 46 g/day) and one of three fast-growing genotypes (ADG: 63 g/day) had lower overall mortality rates than the other two fast-growing commercial genotypes (ADG: 63 g/day), and the intermediate-growing genotype (slaughtered at 60 days of age) had fewer culls and birds found dead from 3 weeks of age until slaughter than the three fast-growing genotypes (Ross 308, Cobb 500, and Hubbard Flex; slaughtered at 42 days of age). The most common causes of found dead birds and culls were yolk sac infections (32%) and lameness (24%). Rayner et al. [7] similarly found that a fast-growing genotype (ADG: 63 g/day) had higher total mortality and a greater percentage of pre-processing culls than two intermediate growing genotypes (ADG: 45 and 49 g/day, respectively). Between the two intermediate-growing genotypes, the genotype with a growth rate of 45 g/day had lower 1 week and total mortality and lower post-mortem inspection rejections than the genotype with a growth rate of 49 g/day. The most common causes of mortality in this study were cellulitis, abnormal color/fevered, hepatitis, perihepatitis, and peritonitis. Slegers et al. [72] found a lower prevalence of mortality in slow- and intermediate-growing genotypes than in fast-growing genotypes, and in slow-growing genotypes compared to intermediate-growing genotypes (ADGs: not reported). Likewise, Forseth et al. [122] examined register data from six years of production in a Norwegian broiler company, and found a higher mortality on-farm and during transport of the fast-growing Ross 308 compared to the slower-growing Hubbard JA787 (ADGs: not reported).

Torrey et al. [123] found that birds with growth rates between 20 and 48 g/day had fewer culls but were more likely to be found dead than birds with growth rates of 50–51 g/day. Within the group of birds with growth rates ranging from 50 to 51 g/day, a genotype with a growth rate of 51 g/day had higher total mortality than a genotype with a growth rate of 50 g/day. Within the group of birds with growth rates ranging from 44 to 48 g/day, more birds from the genotype with a growth rate of 44 g/day were culled than from the genotypes with growth rates of 48 and 46 g/day. A genotype with a growth rate of 44 g/day had more birds found dead than another genotype with a similar growth rate. Across the 16 genotypes, 44% of the culls were due to lameness, with 46% of the cases having fast (69 g/day) and intermediate (55 g/day) growth rates. Castellini et al. [77] classified organic male broilers into three groups based on growth rate. The slowest growing group consisted of Acona (ADG: 17 g/day), leghorn (ADG: 16 g/day), and Cornish × Leghorn (ADG: 23 g/day) genotypes. A Ross genotype represented the fastest growing group (ADG: 55 g/d) while Gaina (25 g/day), Robusta Maculata (ADG: 27 g/day), Kabir (ADG: 29 g/day), and Naked Neck (ADG: 31 g/day) genotypes made up a group of birds with in-between growth rates. The study found that the Ross genotype had the highest mortality, while two of the genotypes in the slowest-growing group (Acona and Leghorn) had the lowest culling rates. There were no significant differences in culling or mortality rates amongst the other slower growing genotypes. In an experimental study comparing six genotypes differing in growth rates, Guinebretière et al. [44] found a higher mortality in the fast-growing Ross 308 genotype (ADG: 61 g/day) from day 11 until slaughter compared to the intermediate-growing Ranger Classic (ADG: 47 g/day). No other differences in late mortality were found between the genotypes examined (intermediate-growing genotypes: Redbro (ADG: 49 g/day), Rustic Gold (ADG: 48 g/day), Hubbard JA787 (ADG: 46 g/d); slower-growing genotype: Hubbard JA757 (ADG: 42 g/day)).

Within studies where genotypes were housed and managed under similar conditions, a clear trend is seen with higher mortality being observed in flocks with higher growth rates. When only growth rate is considered, this trend is not as apparent, likely due to the number of additional management factors that play a large role in mortality rates.

### 3.8. The Impact of Potential Growth Rate on Welfare of Broiler Breeders

The parent stock of broilers, i.e., the broiler breeders, is typically feed-restricted to ensure health and reproductive success [17]. The level of restriction is linked with growth potential, with faster-growing genotypes being restricted to a greater extent. In this section, the breeders are classified based on the growth potential of their offspring (the broilers). Hunger and associated physiological and psychological stress represent a major welfare concern in the broiler breeder population [124,125]. Chronic hunger can lead to the expression of abnormal behaviors indicative of frustration or hunger including increased aggression, injurious pecking, cannibalism, and physiological indicators of stress and immunosuppression [126]. Physiological measures such as blood heterophil to lymphocyte ratios, corticosterone, and dopamine [127] as well as compensatory feed intake [128] change proportionally to the severity of feed restriction, suggesting that slower-growing genotypes that require less severe restriction would be less negatively impacted. Choosing slower-growing genotypes can reduce the severity of feed restriction or may completely eliminate the practice and associated welfare issues.

Dwarf female breeders, such as the Colour Mini or F15 parents from Hubbard, are sometimes used as they have slower growth rates and a smaller body size which results in a lower feed consumption. These genotypes seem to maintain good health and reproductive fitness when fed ad libitum diets [17,129,130]. One study aimed to assess how well three genotypes of broiler breeders tolerate ad libitum feeding [130]. The genotypes were conventional Hubbard breeders, Hubbard dwarf breeders developed for Label Rouge chicken production, and an experimental line of dwarf heavy broiler breeders intentionally selected for improved health and reproductive traits at the expense of growth. Mortality rates were high in the ad libitum-fed conventional breeders, reaching nearly 40% by 40 weeks of age. Egg production in this group was also low, with a high proportion of defective eggs laid. In contrast, the two genotypes with a reduced growth rate demonstrated acceptable reproductive and livability values, though egg production was 10–20% lower in the experimental line. Favorable behavioral measures such as decreased drinking (potentially contributing to improved litter quality), and more time resting have been observed in dwarf genotypes than conventional breeders, which are considered indicators of less hunger experienced [131]. Use of slower-growing genotypes can also promote a more natural behavioral repertoire by allowing them to spend a greater proportion of their day engaged in feeding behavior as they are fed larger quantities of feed which take longer to consume [41]. When comparing signs of chronic stress between three slower-growing female and two intermediate-growing male genotypes with varying growth potential it was found that genotypes requiring lower feed restriction exhibited lower signs of feeding frustration and had better weight uniformity [132].

Feed restriction in general is associated with the development of unwanted behaviors such as stereotypic pecking, aggression, feather pecking, and cannibalism [125,126,133]. Studies addressing the direct link between growth rate and the development of abnormal behaviors are limited, but it may be hypothesized that the greater extent of feed restriction required for rearing fast-growing genotypes may contribute to greater occurrences of behaviors associated with hunger, frustration, and competition for feed. These behaviors may include stereotypies (such as polydipsia and pacing) and damaging behaviors (such as aggression and cannibalism). In a study comparing a Hubbard dwarf breeder genotype with a dwarf heavy broiler breeder selected for improved livability at the partial expense of growth and a fast-growing Hubbard broiler breeder, it was found that the dwarf heavy broiler breeder and the conventional broiler breeder pecked at the empty feeder for 10–13% of the time, compared to only 4% in the slow-growing dwarf breeder, indicating less hunger and frustration in the latter [134].

In summary, breeders with lower growth potentials do not require as severe levels of feed restriction as their faster-growing counterparts. Based on the available literature, it appears that genotypes with slower growth potentials display lower levels of behavior associated with hunger and frustration. For example, genotypes with lower growth potential spend less time drinking, which may contribute to improved litter condition, and spend more time resting. Broiler breeders with slower growth potentials also display better weight uniformity and have lower mortality rates.

## 4. Conclusions

It is clear from the available literature that genotypes with substantial differences in their growth rates have significant differences in welfare indicators, with those having slower growth showing improved welfare. However, making inferences on the impact of minor changes in growth rate is more challenging, as there are fewer available studies and of those, many classify genotypes into broad categories for analyses, making direct comparisons between specific growth rates difficult. Further, even between genotypes with similar growth rates there may be differences in body conformation, behavioral attributes, etc., which could affect their welfare, complicating comparison purely of their growth rates. In addition, genotypes change with each breeding cycle and results found several years ago might no longer be directly applicable.

Genotypes with faster growth rates are more likely to suffer from poor leg health and compromised walking ability. Experimental trials and surveys of commercial flocks consistently find higher frequencies of worse gait scores, indicating compromised walking ability which can impact their ability to perform behaviors and access resources. Prevalence of musculoskeletal system disorders is also greater in genotypes with faster growth rates. Walking ability and the occurrence of varus-valgus deformities have been shown to be impacted by even minor differences in growth rates, with differences of as little as 4–5 g/day significantly impacting the proportion of birds with the different gait scores. When it comes to the prevalence of contact dermatitis, the evidence on the impact of minor differences in growth rate is inconsistent.

The greater freedom of movement associated with slower growth means that genotypes with slower growth rates are generally more active and have a greater ability to perform motivated behaviors and to access a covered veranda or an outdoor range. The literature consistently shows that faster growth rates are associated with birds spending more time sitting and less time walking, foraging, interacting with their environment, and engaging in behaviors associated with positive affective states. In addition, genotypes with slower growth show higher use of outdoor ranges. When comparing ability to perform specific behaviors, it is less clear what impact a small change in growth rate could have on welfare. Generally, studies indicated that genotypes with similar growth rates displayed similar behavioral patterns, and where differences did exist the link with growth rate was not consistent.

Selection for fast growth rates and larger breast muscle size has led to higher prevalence of breast muscle myopathies and an increased susceptibility to heat stress in fast-growing genotypes. Generally, genotypes with higher growth rates experience more negative impacts from exposure to heat stress. Even minor differences in growth rates (around 5 g/day) appear to affect susceptibility to heat stress, with high temperatures leading to the least weight reduction, lower mortality and lower body temperatures in the slower growing broilers. There is a lack of studies on prevalence of breast muscle myopathies comparing genotypes with minor differences in growth rates but restricting growth rate within a genotype by 5 g/day through feed restriction has been shown to result in a reduction in white striping in the breast muscle.

Broilers with slower growth rates experience lower mortality rates than their fast-growing counterparts, likely in part due to their improved walking ability, lower rates of contact dermatitis (and thus risk of secondary infections), improved cardiovascular health, and more robustness to heat stress. Results on how small differences in growth rate impact mortality are inconsistent, with some evidence suggesting that small reductions in growth rate (1–4 g/day) can lower mortality rates, whereas other studies have found no effect or the opposite effect.

Overall, when comparing broiler genotypes with substantial differences in their growth rates, those with slower growth rates consistently show improved welfare. The few existing studies that compared genotypes with minor differences in growth rate predominantly found that the slowest growing of the genotypes studied showed improved welfare, indicating that while other factors may have an impact, even minor reductions in growth rate can result in improved welfare.

## Figures and Tables

**Table 1:** Average prevalence (%) of gait scores in each of the Bristol Gait Score categories, displayed for various broiler genotypes and arranged according to study (separated by thin vertical lines). In addition, the genotype (if known), growth rate (GR; g/d), stocking density (SD; kg/m^2^), housing conditions, and weeks of age when scoring was conducted are provided. Modified from Schuck-Paim et al. [45].

					Gait Score						
Genotype	GR: (g/d)	SD: (kg/m^2^)	Housing	Wks.	0	1	2	3	4	5	Source
Ross 308	31.8–50.8	36	Commercial	4.40	4.80	31.40	39.00	22.70	2.00	0.20	[46]
Ross 308	35.1–48.2	15–33	Commercial	4.1	3.00	44.00	34.00	16.00	2.00	1.00	[38]
Ranger Gold	38	21	Commercial	7.5	7.68	65.73	22.09	3.97	0.36	0.18	[43]
Hubbard JA757	46	18.7	Experimental	7	18.50	46.30	31.30	3.80	0.91	0.09	[6]
Hubbard JA757	46	21	Experimental	8	7.40	33.50	41.80	17.30	0.91	0.09	
Unknown	44	30	Quasi-commercial	7	13.90	63.10	22.50	0.70	0.45	0.04	[7]
Unknown	47	30	Quasi-commercial	7	5.70	51.00	40.90	2.60	0.71	0.07	
Unknown	47	34	Quasi-commercial	7	6.10	52.90	37.50	3.50	0.56	0.06	
Devonshire Red	41	unknown	Commercial	8	8.60	39.10	41.40	10.80	0.30	0.00	[47]
Ross 708 (*n* = 14), Ross 308 (*n* = 2), Hubbard (*n* = 2)	52	26	Commercial	6	0.80	11.20	57.50	25.20	4.20	1.20	
Ross (*n* = 17), Cobb (*n* = 1)	54	44	Commercial	6	1.00	16.00	57.00	22.00	3.00	1.00	
Ranger Gold	43–45	38	Commercial	7	6.90	60.84	25.62	5.91	0.49	0.25	[8]
Rustic Gold	47–51	38	Commercial	6	5.15	58.57	32.15	3.64	0.36	0.13	
Ross 308	62–63	40	Commercial	4.6	4.58	53.91	35.51	5.35	0.57	0.09	
Hubbard JA757 and CJA57	35	21	Commercial	8–9	61.90	28.30	7.40	1.80	0.50	0.10	[5]
Ross 308	63	40	Commercial	4.6	22.60	41.10	30.80	4.70	0.60	0.10	
Redbro	53	30	Commercial	7	64.00	29.00	7.00	0.00	0.00	0.00	[48]
Ross 308	65	30	Commercial	6	46.00	41.00	11.00	1.00	1.00	0.00	
Ross	59–63	28–40	Commercial	5.6	0.10	0.60	16.40	68.30	14.00	0.60	[49]
Ross 308	71	39.5	Commercial	6	0.00	5.80	74.40	19.00	0.60	0.20	[32]

**Table 2:** Percentage of total culls, total found dead, and total mortality for broiler genotypes of various growth rates, ordered according to study (separated by thin vertical lines). Genotype, growth rate (GR: g/d), and stocking density (SD: kg/m^2^) are provided when known.

Genotype	GR: (g/d)	SD: (kg/m^2^)	Total Culls (%)	Total Found Dead (%)	Total Mortality (%)	Source
Hubbard JA787	“Slower-growing”	33.8	NA	NA	1.84	[122]
Ross 308	“Fast-growing”	32.8	NA	NA	3.84	
Unknown	17.5	28.1	2.08	1.04	3.13	[123]
Unknown	41.2	31.7	0.38	1.52	1.89	
Unknown	42.0	34.3	1.14	2.27	3.41	
Unknown	42.0	28.1	0.19	2.08	2.27	
Unknown	42.0	28.1	0.57	3.79	4.36	
Unknown	42.9	28.1	0.76	0.76	1.52	
Unknown	44.7	28.1	0.19	2.27	2.46	
Unknown	46.7	34.3	0.38	1.70	2.08	
Unknown	47.7	34.3	0.76	1.14	1.89	
Unknown	47.7	31.7	0.76	0.95	1.70	
Unknown	48.8	34.3	1.33	1.70	3.03	
Unknown	50.0	31.7	1.33	2.27	3.60	
Unknown	52.5	31.7	0.76	1.14	1.89	
Unknown	56.8	32.0	0.38	1.52	1.89	
Unknown	58.3	32.0	1.33	2.46	3.79	
Unknown	61.8	32.0	0.57	2.27	2.84	
Hubbard JA757	42	37, 29	NA	NA	12.5, 8.3	[44]
Hubbard JA787	46	37, 29	NA	NA	4.9, 5.7	
Ranger Classic	47	37, 29	NA	NA	4.0, 3.4	
Rustic Gold	48	37, 29	NA	NA	4.8, 4.0	
Redbro	49	37, 29	NA	NA	5.5, 6.2	
Ross 308	61	37, 29	NA	NA	5.1, 5.4	
Hubbard JA757	46	21.3	2.33	2.79	5.12	[6]
Unknown	63	21.3	2.62	4.76	7.38	
Unknown	63	21.3	6.40	4.26	10.66	
Unknown	63	21.3	5.66	5.19	10.85	
Mixed	“Slow”	Varied	NA	NA	1.9	[72]
Mixed	“Medium”	Varied	NA	NA	2.0	
Mixed	“Conventional”	Varied	NA	NA	3.2	
Leghorn	16	Unknown	0	4	4	[77]
Acona	17	Unknown	0	3	3	
Cornish × Leghorn	23	Unknown	2	4	6	
Gaina	25	Unknown	2	3	5	
Robusta Maculata	27	Unknown	2	4	6	
Kabit	29	Unknown	3	6	9	
Naked Neck	31	Unknown	2	5	7	
Ross	55	Unknown	5	14	19	
Unknown	45	30	0.90	1.21	2.11	[7]
Unknown	49	30	1.49	1.71	3.20	
Unknown	49	34	1.22	1.37	2.59	
Unknown	63	34	3.12	3.11	6.23	

## Data Availability

Not applicable.
